# Breakfast: To Skip or Not to Skip?

**DOI:** 10.3389/fpubh.2014.00059

**Published:** 2014-06-03

**Authors:** Tanya Zilberter, Eugene Yuri Zilberter

**Affiliations:** ^1^Infotonic Conseil, Marseille, France; ^2^School of Psychology, University of Glasgow, Glasgow, UK

**Keywords:** dietary health education, breakfast skipping, overnight fast, late eating, health risks, intermittent fasting, meal timing, calorie intake

Human eating behaviors are often non-homeostatic, and thus unlike homeostatic behaviors, they are not exclusively reliant on rigid brain mechanisms, but heavily depend on psychological, sociocultural, and educational factors as well. A clear understanding of the mechanisms and consequences of various eating behaviors is necessary for giving comprehensive educational guidance. However, recommendations regarding breakfast (BF) eating behavior are perhaps the most peremptory yet scientifically (especially metabolically) groundless health guidelines, and thus the widely accepted notion of BF as the most important meal of the day has been called into question. In a recent meta-analysis, Brown et al. (1), not arguing with the established link between obesity and BF behavior, concluded: “*The current body of scientific knowledge indicates that the proposed effect of breakfast on obesity is only presumed true*” (p. 1298). The authors state that numerous articles demonstrating negative metabolic effects of skipping BF have yet to establish a causal relationship due to a lack of probative value and that the major obstacle in establishing causality is neglecting the possible confounding factors.

In this opinion paper, we suggest that BF is just another meal, rather than the “most important meal of the day” as is commonly believed and that prolongation of overnight fast, which depends not only on timing of BF but also on timing of the last meal of the day, can be beneficial.

## Definition

A significant barrier to advances in the study of BF behaviors is the lack of a common language. It is often discussed that there is a fundamental difficulty in comparing different results due to lack of common definitions for both eating BF and skipping BF [e.g., Ref. (2, 3)]. Currently, the definition of BF continues to vary between studies, although the definition by Timlin and Pereira (4): “*first meal of the day, eaten before or at the start of daily activities (e.g., errands, travel, work), within 2 h of waking, typically no later than 10:00 in the morning, and of an energy level between 20 and 35% of total daily energy needs*” is accepted as an academic standard. Of significance for this discussion is to note that the above definition does not specify the duration of the overnight fast.

## Duration of Overnight Fast

**Figure 1:**
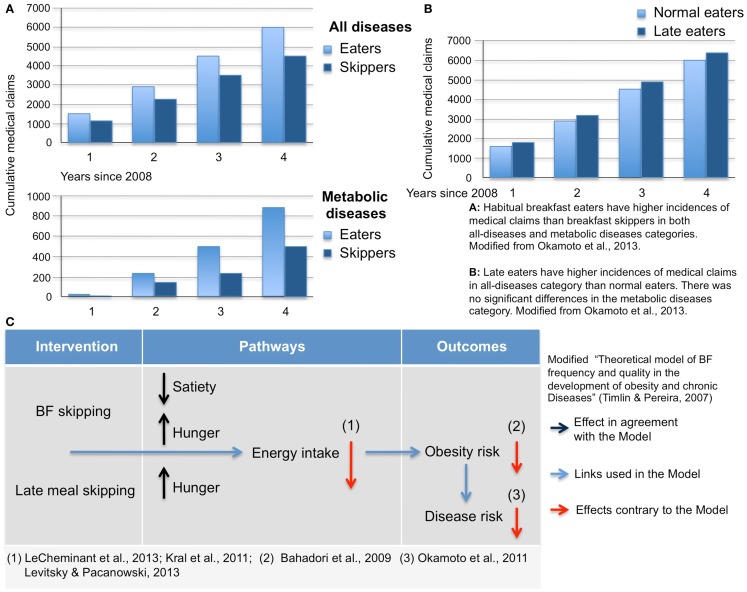
**Habitual breakfast eaters (A) and late eaters (B) have higher incidences of medical claims than breakfast skippers and early eaters**. Modified from Ref. (7). **(C)** Amendment to the “*theoretical model of BF frequency and quality in the development of obesity and chronic diseases*” (4).

The duration of overnight fast is rarely specified in studies comparing effects of eating versus skipping BF. As a result, an important parameter, which can significantly influence the metabolic consequences of eating behaviors are neglected in the majority of studies resulting in wrong conclusions (e.g., 4; Figure 1C).

Consider, for example, two BF-eaters having their last meals at midnight; one then consumes BF at 5:00 a.m. (5-h overnight fast) and the other consumes BF at 10:00 a.m. (10-h overnight fast). Both are considered “BF-eaters” in the majority of studies, but the difference in overnight fast durations makes them metabolically distinct from each other. Similarly, “BF-eaters” who have an earlier dinner (last meal) at 7:00 p.m. followed by BF at 10:00 a.m. have an overnight fast of 15 h, which can be metabolically similar to “BF-skippers” who had a later dinner (midnight) and skipped BF altogether and ate their first meal at 3:00 p.m. (same 15-h overnight fast). The important point here is that due to the neglect of this important parameter in BF eating behavior studies, it challenges the validity of their findings and the interpretations thereof.

The above point also shines light on potential contradictions in eating behavior literature. For example, although eating late (practicing late dinners and/or night-time meals) is considered a bad habit and eating BF is considered healthy, they may both have similar duration of overnight fasts and thus may result in similar metabolic profiles. It is well known that “late eaters” have a problem with losing weight (5, 6) and have overall poorer health conditions [Figure 1B, Ref. (7)]. Both late eating and BF skipping are usually listed together as risk factors for unhealthy metabolic profiles [e.g., Ref. (8, 9)] but solid evidence exists only for consequences of late eating.

Late or night-time eating was found to be linked to multiple eating behavior pathologies, such as night-time hunger, body image distortions, and mood disorders (10), as well as elevated blood levels of insulin and glucose characteristic for metabolic syndrome (11). Timing of eating behavior has both circadian and non-circadian aspects (12). The non-circadian aspect (that is, 24-h caloric intake) allows for a comparison between the effects of skipping BF and excluding late eating, in terms of duration of the overnight fast. When late eating is excluded, as it was in a randomized crossover design study (13) where no eating was assigned from 7:00 p.m. to 6:00 a.m. while *ad libitum* eating was allowed during the rest of the day, the subjects consumed 244 fewer kilocalories regardless of their food choice. The calorie intake factor is one of the most important in determining the metabolic consequences of meals and eating behaviors.

The model proposes that regular BF consumption can lead to increased satiety and decreased hunger [which is in agreement with data of Kral et al. (14) and Levitsky and Pacanowski (15)]. However, research results of Levitsky and Pacanowski (15) do not support this claim. The lower risk of chronic disease suggested in the Model is in disagreement with data depicted in Figures 1A,B.

## Breakfast Skipping: What Do We Really Know?

To investigate the link between BF skipping, BMI, and risks of obesity, a closer look at caloric intake may be used as a tool to establish whether or not there is any causality between them. Calorie intake is considered the major determinant of BMI [e.g., Ref. (16)]. It is routinely stated that skipping BF is overcompensated with increased energy consumption later during the day. Indeed, many authors state that skipping BF results in increased BMIs [e.g., Ref. (17, 18)] although (as mentioned in the introduction) no causality has been established. Additionally, even the very existence of such a link is questioned. While skipping BF has been linked to higher risk of obesity in the UK (19), Hong Kong (20), and the USA (21), no such link was observed in Australia (22), Portugal (23), and Saudi Arabia (24).

A link between skipping BF and obesity is constantly being challenged and in many studies, a lack of this link was repeatedly demonstrated (24–32). In fact, the exact opposite link was recently demonstrated: in a large cohort, 4-year long study based on Japanese insurance statistics (7), the accumulation of newly diagnosed diseases was plotted against various lifestyle-related behaviors. Self-reported BF-skippers had a lower incidence of all diseases (including metabolic diseases) as compared to BF-eaters. In the same study, a link between late eating and poorer health was demonstrated (Figure 1A).

## Controlled Studies of Energy Balance

The supposed disadvantages of skipping BF have not been supported by recent controlled studies. Kral et al. (14) directly demonstrated that skipping BF caused an increase in hunger levels but not in a overcompensation of calorie intake later in the day. On the contrary, Gonzalez et al. (33) found that daily energy and fat intakes were reduced with BF omission. Similarly, Levitsky and Pacanowski (15) showed that, although skipping BF significantly increased hunger ratings at lunchtime, food intake at lunch were not increased. Moreover, skipping BF resulted in a net energy deficit of about 400 kcal a day comparing to BF eating group. Levitsky and Pacanowski mention that 25% of Americans now regularly skip BF in order to lose weight compared to 14% in 1965, despite aggressive campaigns labeling skipping BF as one of the most harmful eating behaviors. The caloric intake reduction due to skipping BF may offer an explanation for why this practice for successful weight loss works and is on the rise. A similar mechanism of caloric intake reduction due to skipping BF is present due to abstaining from late eating, as demonstrated by LeCheminant et al. (13).

Another component of energy balance is energy expenditure. Recently, it was shown that skipping BF did not affect 24-h energy expenditure, resting metabolic level, or food-induced thermogenesis (34). This seems to contradict the description of BF-skippers as more sedentary, having decreased energy expenditure [e.g., Ref. (35)]. However, it cannot be excluded that a third factor exists which underlies both skipping BF behavior and sedentary lifestyle (36). In a recent Danish longitudinal study of 8- to 11-year-old children, examining the causality between being overweight and physically inactive, a causality was indeed found, but the exact opposite to what is commonly believed, “*that adiposity is a better predictor of physical activity and sedentary behavior changes than the other way around*” [(37), p. 1]. If adiposity is, in fact, the reason for physical inactivity, then by the same token, BF skipping may be a result of being overweight, and not the other way around.

## Indirect Evidence of the Potential Benefits of Skipping Breakfast

Comparing to evidence from the broader field of nutrition, we can investigate a potential overlap, which can shine light on the potential benefits of skipping BF. In terms of meal timing, skipping BF is similar to intermittent fasting (38), however, it is seldom looked at from this point of view. One of metabolic effects of intermittent fasting is intermittent ketosis known for its appetite suppression effect (39–41) resulting in voluntary calorie reduction [e.g., Ref. (42)]. BF skipping and exclusion of late eating, as described above, also result in reduction of voluntary calorie intake (15, 33). Calorie restriction has been shown to have profound metabolic benefits including neuroprotective, anti-aging, and anti-inflammatory [see Ref. (43) for review]. Furthermore, Mattson and colleagues showed in rodents that intermittent fasting had more metabolic benefits than permanent calorie restriction (44), thus skipping BF may be more beneficial than traditional restrictive dieting.

## Conclusion

Given body of evidence reviewed in this opinion article, it is reasonable to suppose that skipping BF could be as metabolically beneficial as excluding late eating, as well as stress the importance of the overnight fast. Perhaps it does not matter which of the daily meals – the first or the last – is omitted as long as at least once in a while, an inter-meal interval is long enough to allow the state of ketosis to initiate lipolysis and lower calorie intake, thus decreasing the risk of obesity and its comorbidities.

## Conflict of Interest Statement

The authors declare that the research was conducted in the absence of any commercial or financial relationships that could be construed as a potential conflict of interest.
